# SARS-CoV-2 screening: effectiveness and risk of increasing transmission

**DOI:** 10.1098/rsif.2021.0164

**Published:** 2021-07-21

**Authors:** Jordan P. Skittrall

**Affiliations:** ^1^ Department of Applied Mathematics and Theoretical Physics, University of Cambridge, Centre for Mathematical Sciences, Wilberforce Road, Cambridge CB3 0WA, UK; ^2^ Cambridge Clinical Microbiology and Public Health Laboratory, Box 236, Addenbrooke’s Hospital, Cambridge CB3 0WA, UK; ^3^ Department of Infectious Diseases, Cambridge University Hospitals NHS Foundation Trust, Addenbrooke’s Hospital, Hills Road, Cambridge CB2 0QQ, UK

**Keywords:** COVID-19 pandemic, SARS-CoV-2, mass screening, infection transmission

## Abstract

Testing asymptomatic people for SARS-CoV-2 aims to reduce COVID-19 transmission. Screening programmes’ effectiveness depends upon testing strategy, sample handling logistics, test sensitivity and individual behaviour, in addition to dynamics of viral transmission. The interaction between these factors is not fully characterized. We investigated the interaction between these factors to determine how to optimize reduction of transmission. We estimate that under idealistic assumptions 70% of transmission may be averted, but under realistic assumptions only 7% may be averted. We show that programmes that overwhelm laboratory capacity or reduce isolation of those with minor symptoms have increased transmission compared with those that do not: programmes need to be designed to avoid these issues, or they will be ineffective or even counter-productive. Our model allows optimal selection of whom to test, quantifies the balance between accuracy and timeliness, and quantifies potential impacts of behavioural interventions. We anticipate our model can be used to understand optimal screening strategies for other infectious diseases with substantially different dynamics.

## 1. Background

Repeatedly screening asymptomatic individuals for SARS-CoV-2, with the aim of isolating infected people and thereby reducing transmission, has been undertaken in hospitals [1], institutions [2,3], professional sports leagues [4] and the White House. It has been undertaken at town and city level [5,6], with the aim of expansion to national level [7]. This reduction in transmission aims not only to save lives but also to permit continuance of activities that would otherwise be halted as part of disease control efforts.

The success of such screening in reducing infections does not rely only upon screening frequency, test sensitivity and viral shedding profiles. It encompasses every element of the screening process, from those affecting whether people with infection undergo screening, through the speed with which screening can inform people they are infectious, to the actions people take on learning they are infectious. Understanding the contributions from and interactions between these elements is key to designing an effective screening programme. Crucially, it is key for avoiding a programme that loses effectiveness or even increases infection rates by allowing the wrong circumstances to come together.

We have derived an expression for the proportion of infections averted by a screening programme. Our expression accounts for real-world engagement with screening and the time taken to process samples. We show that in realistic situations, screening (with isolation following a positive screen) alone results in only a modest reduction in infections. When the presence of screening results in a relaxation of precautions taken by those with minor symptoms, we show that this combination can result in an overall increase in infections compared with no screening. We demonstrate that the success of screening depends upon a rapid turnaround of tests. As a result, we show that a screening programme running comfortably under capacity is more successful than one that pushes capacity and generates backlogs. Our derived expression can be used to compare the effectiveness of proposed testing strategies in complex scenarios where there are different infection rates and different test availabilities—such situations might occur in a small screening programme, for example in a hospital with ward-to-ward variation in infection, or in a large programme, for example in a national programme with city-to-city variation in infection and in laboratory locations.

## 2. Results

We began by considering two different screening scenarios. The first scenario is an ideal (maximum impact) scenario. In this scenario, screening is performed daily with a high-sensitivity test. Test turnaround is rapid. All those eligible for screening present on every occasion, and all those with positive screens immediately isolate. The second scenario is a realistic scenario for mass population screening. In this scenario, screening is performed weekly with a high-sensitivity test. Samples for testing must be transported by courier from the sampling site to the testing laboratory. Test turnaround follows the distribution of real turnaround times sampled from a large laboratory operating within capacity. There is attrition reducing those eligible who present for screening and those who isolate following positive screens, similar to the attrition observed in other screening programmes. The difference between the two scenarios is marked. In the ideal scenario, we estimate screening alone can eliminate 70% of onward transmissions (95% confidence interval 66–74%). In the realistic scenario, the proportion of transmissions eliminated reduces to 7% (95% confidence interval 5–11%).

**Figure 1.**
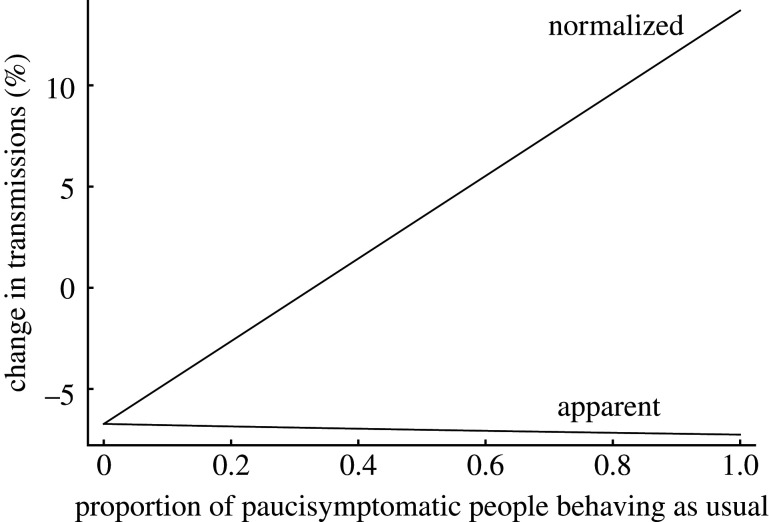
Behaviour changes in people with minor symptoms may negate the effect of screening. Model output where the proportion of those developing minor symptoms (paucisymptomatic people) behaving as usual, rather than isolating, is varied from 0 to 1, with parameters otherwise as in our realistic scenario. There are two ways of viewing the effect of screening. Firstly, one can just consider the effect of screening and isolation itself, so that as fewer people isolate before being screened, there are more transmissions to interrupt and so screening appears to stop more transmissions (‘apparent’ change in transmissions line, with number of transmissions in absence of screening as denominator, showing estimated change in transmissions varying from −6.7% when all paucisymptomatic people isolate, to −7.3% when all paucisymptomatic people behave as usual). Secondly, one can consider changes in behaviour also to be part of the impact of screening: in this case, the effect is the change in transmissions caused by an increase in the proportion of paucisymptomatic people behaving as usual from a fixed proportion (which we define to be zero, i.e. all paucisymptomatic people isolating) *and* then screening and isolation of those not already isolating (‘normalized’ change in transmissions line, with number of transmissions in the absence of screening when all paucisymptomatic people isolate as denominator, showing estimated change in transmissions varying from −6.7% when all paucisymptomatic people continue to isolate, to +13.7% when all paucisymptomatic people behave as usual). The combined effect is at best a reduced effectiveness in screening, and at worst an increase in the number of transmissions. (Note that the figure can be redrawn with the ‘normalized’ line representing the change from a different fixed proportion of paucisymptomatic people behaving as usual prior to the introduction of screening, but that as long as screening causes the proportion to increase, the overall result will still hold.)

We next considered what happens if the presence of screening reassures those with minor symptoms, so that instead of isolating they continue with their daily lives. We divided our infected population into three categories: those who never display any symptoms of infection and always continue with their daily lives, those who display typical symptoms and isolate as soon as these symptoms manifest, and those who display minor symptoms and variably reduce their contact with others when such symptoms manifest. If, instead of isolating, those with minor symptoms behave as usual, the total number of transmissions increases, and because on average an infected person remains infectious for longer (having not isolated), the proportion of transmissions eliminated by screening increases. However, because screening does not identify all the additional individuals in the population with minor symptoms but behaving as usual, the net result is a relative increase in the number of transmissions. If the behaviour change is seen in a sufficiently high proportion of those with minor symptoms, the net result of the screening programme can be an absolute increase in transmissions compared with no screening programme, even though the programme appears to be more successful (figure 1).

**Figure 2.**
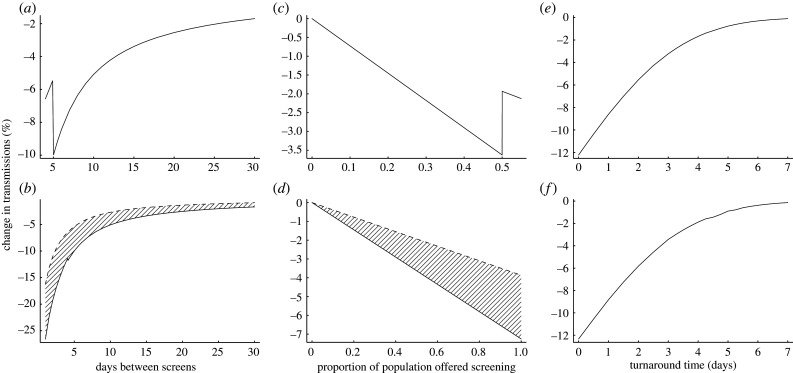
Turnaround time strongly impacts the success of screening. (*a*) Impact on transmissions of shortening the interval between screening tests, until, at a 5-day interval, testing capacity is overwhelmed with an impact on turnaround time. The increased turnaround time results in delays in isolating infectious people and a drastic loss in ability to prevent transmissions. (*b*) Comparison (hatched region) between the effects of normal (solid line) and impaired (dashed line) laboratory turnaround times on transmission reduction, for varying screening intervals. (*c*) Impact on transmissions of offering weekly screening to an increasing population proportion until, at 50%, testing capacity is overwhelmed with an impact on turnaround time. As in (*a*), the resultant delays to isolation cause a drastic loss in ability to prevent transmissions. (*d*) Comparison (hatched region) between the effects of normal (solid line) and impaired (dashed line) laboratory turnaround times on transmission reduction, for varying proportions of the population offered screening. (*e*) Impact of turnaround time on transmission reduction. Here, rather than being a distribution, total turnaround time from sampling to action on a positive result takes a single value, which is varied. (*f*) As (*e*) but using reported RNA detection rates from the literature [8] rather than assuming the probability of detection scales with infectiousness. This shows the results are not an artefact of assuming detecting infection is more likely in more infectious individuals. Our realistic model parameters are used where not otherwise stated.

Following this, we considered the impact of testing turnaround times on the ability of screening to reduce viral transmission. In general, one would expect a greater number of screening tests to be able to detect a greater number of infections, and therefore to yield a greater reduction in transmissions. However, when the number of tests requested exceeds a laboratory’s capacity, a backlog develops with consequent increase in turnaround time. This effect was seen in English laboratories at the end of April 2020, when a policy of testing large numbers of asymptomatic people in residential facilities was implemented. We modelled the effect of exceeding laboratory capacity using turnaround time data from April to June 2020 in our regional clinical microbiology and public health laboratory in Cambridge, England (figure 2). Our output shows that reliably keeping laboratory demand slightly below capacity results in a greater reduction in transmissions than when capacity is exceeded. We then proceeded to consider the general effect of laboratory turnaround time on the ability of screening to reduce viral transmission. Our output shows that the extent of transmission reduction depends strongly upon turnaround time.

**Figure 3.**
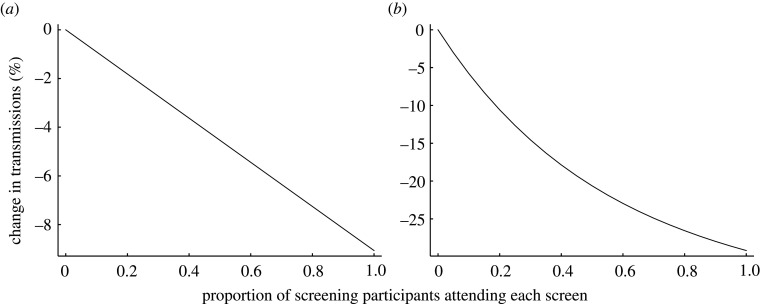
Diminishing returns with increased uptake for short screening intervals. Model output showing change in transmissions as the proportion of those who attend each offered screen (from those who engage in screening at least once) changes. (*a*) Weekly screening interval. (*b*) Daily screening interval. Model parameters are set as for our realistic scenario, except for the proportions attending each screening, and the screening interval in (*b*).

A full consideration of screening effectiveness takes into account individual engagement with screening. We considered this in our model, via the proportion of those offered screening who ever take it up, the proportion of those who attend each screening event, and the proportion who isolate when asked. The first and last of these have a linear effect on testing effectiveness (electronic supplementary material, figure S1). For a weekly screening interval, the second is also approximately linear, reflecting that this screening interval only gives one opportunity to prevent most transmission. With more frequent screening intervals, the impact on transmissions of increased per-screen uptake becomes nonlinear, with diminishing returns as the uptake is higher (figure 3).

**Figure 4.**
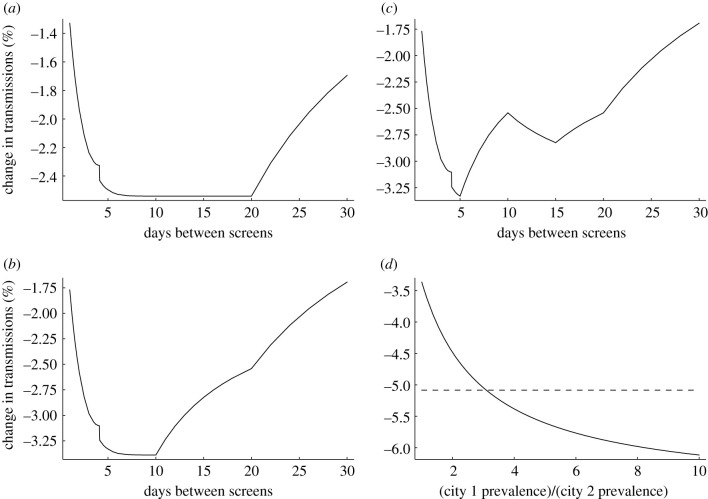
Screening in more complex scenarios. (*a*) Scenario where testing capacity is sufficient to offer screening to the whole population every 20 days (taking non-attendance into account), and offering of tests is managed so as not to exceed capacity (a smaller proportion of the population is screened if testing is offered more frequently than every 20 days). The proportion/frequency combination does not affect testing impact, except at the extremes where tests are not all used on potentially infected individuals (right-hand side of plot: testing frequency is so low that some tests are unused; left-hand side of plot: testing frequency is so high that some people offered tests have already tested positive and isolated in a preceding testing round). Note that the very small discontinuity of approximately 0.1% near the 4-day interval in this and subsequent panels is a numerical artefact arising from the code implementation of the calculation; there is no reason to expect such a discontinuity in a real-world setting. (*b*) As (*a*) but with the population structured: half the population has twice the risk of being infected as the other half of the population. The higher risk portion of the population is prioritized for screening, with leftover tests at the chosen screening rate offered to the lower risk portion. In this scenario, the greatest reduction in transmissions occurs when all tests are offered to the high risk portion. (*c*) As (*b*) but half of each portion of the population are healthcare workers in contact with vulnerable patients, so are always screened before others, i.e. the screening priority is higher rate healthcare workers, lower rate healthcare workers, higher rate others, lower rate others. Screening all those in contact with the vulnerable more often (10 day screening interval) is possible at a cost of reduced efficacy of screening in the overall population. (*d*) Scenario in which there are two identical cities with two identical laboratories (realistic testing scenario), save that the first city has an infection prevalence greater than the second city. Laboratory capacity is sufficient to offer screening to everybody every 10 days. Each city’s laboratory can be used separately to offer screening to its local population (dashed line). Alternatively, both laboratories can be used to screen every 5 days those in the city with higher infection prevalence, but with an additional two-day turnaround delay for samples sent between cities (solid line). The more effective strategy depends upon relative infection rates in the two cities.

Because our model takes infectivity and testing distributions as input, limited only by the tractability of the resultant numerical integration, it can be used to predict the impact of different testing strategies in complex scenarios where rates of infection, access to testing or uptake vary within a population (figure 4). This allows us to, for example, consider the impact of a programme using rapid near-patient tests daily, where such tests have lower sensitivity than laboratory-based nucleic acid amplification testing [9]. Assuming total and per-test uptake remains the same (increased frequency balanced by increased convenience), our model predicts a reduction in transmissions from such a programme between 25% for self-administered tests and 32% for laboratory staff-administered tests (95% confidence intervals 22–28% and 29–35%, respectively), compared with the 7% reduction (95% confidence interval 5–11%) from a centralized mass testing programme.

Our model is sufficiently robust to changes in modelling assumptions regarding viral transmission dynamics to make predictions that can be used in practice (electronic supplementary material, table S1).

## 3. Discussion

Ultimate control of the COVID-19 pandemic is unlikely prior to deployment of effective vaccines. Screening and isolation acts as a bridge to this goal, saving lives and permitting some resumption of economic and social activity. It must, however, be recognized that unintended behavioural responses to screening may not only remove the opportunity to resume desired activity, but indeed may make disease transmission worse than if no screening had occurred.

Pursuing centralized testing strategies may rapidly increase testing capacity. However, the critical impact of turnaround time on testing effectiveness means that such strategies must have highly streamlined logistics chains to retain effectiveness. If rapid centralized turnaround of tests is not possible, then less accurate, localized testing may be more effective. As with the historical introduction of screening programmes, in SARS-CoV-2 testing it is crucial we move from simply counting numbers of tests, to more sophisticated measures of their effectiveness.

Engagement with screening substantially impacts success and must not be taken for granted [10]. A holistic approach considering the social, economic and political impacts, acknowledging the incidence of false-positive results and balancing their impact, and combined with good communication, is therefore a key part of a successful programme [11,12]. Testing strategies that employ confirmatory testing to reduce false-positive rates need clear protocols for communicating with those awaiting confirmatory testing, to retain the benefit of earlier isolation.

Our results can only be as accurate as the estimates on which our model is based (for example, of test sensitivity and of duration of asymptomatic viral shedding). We note that the exact values of these estimates depend upon underlying data that may depend upon population characteristics and are sometimes sparse, but would anticipate our numerical results to covary with such estimates in a way that allows comparison between different scenarios. The heterogeneity of the underlying estimates makes the provision of meaningful confidence intervals for derived results difficult. It is important to understand that the main value of our results comes from the insight yielded into which strategic decisions to take when optimizing a screening programme, rather than providing a before/after recipe of the effect of introducing screening de novo. However, where inaccurate underlying estimates limit the accuracy of our predictions, our model highlights what additional information is required. (Our model is also limited by numerical accuracy in the calculations, but the underlying estimates represent a greater uncertainty.) In any case, our model demonstrates the impact of the different modifiable factors in a screening programme, enabling policymakers to prioritize those that give most benefit. The code we have provided (electronic supplementary material, code S1) can be updated with revised estimates as more data become available.

Our study does not account for the potential impact on transmission rate of isolating contacts of those who screen positive. When screening itself does not identify all those infected but not isolating, its impact can be potentiated by isolating contacts or by targeting contacts for additional screening as a higher risk group (similar to the scenario in figure 4*b*). The impact of contact isolation on transmission is highest at an intermediate screening impact, where screening is sufficiently impactful to identify many contacts to isolate, but not sufficiently impactful to have prevented most infections by itself (in the extreme case that screening stops all infection, no contact tracing is necessary). As screening identifies more infectious individuals, both the scale of contact tracing required and the impact from isolating non-infectious contacts increase. As long as there is not competition for resources, the relative efficacy of screening is not impacted by contact tracing (although if contact tracing is successful, there should be fewer infectious individuals to screen), so the approaches can be considered separately.

Our work allows policymakers to design SARS-CoV-2 screening programmes to maximize impact in reducing transmission. While the data used pertain to SARS-CoV-2, the underlying methodology is applicable to any transmissible disease, and can therefore be applied to other epidemics and pandemics.

## 4. Methods

Our model considers the total infectivity of a population over a period of time, and the proportion of this infectivity that is averted by screening and isolating infectious people. The only major assumption underlying the model is that those being offered screening are representative of the overall population in terms of potential infectiousness and behaviour: if this assumption cannot be met, but the population can be broken down into sub-populations where the assumption can be met, then the model can still be used. In particular, we note that the model can be used when the population is not static (individuals entering and leaving the population), but in small populations where there is substantial temporal clustering of both cases of infection and screening events, the actual proportion of transmissions averted may deviate substantially from the estimate.

Infectivity is deemed to end naturally at the end of asymptomatic viral shedding, or when an individual develops symptoms and isolates from others. We construct the model by starting with a simplified screening scenario. We then add more realistic components stepwise until we have reached the final model. Ultimately, the model takes as input distributions of virus shedding duration, infectivity, intervals between tests, testing turnaround times, detection probabilities, and population engagement with screening.

We begin by considering the scenario represented in electronic supplementary material, figure S2. Asymptomatic/presymptomatic shedding lasts for a duration *m* ∈ *M*. It begins during a screening interval of duration *j* ∈ *J* (in principle, successive screening interval durations can be drawn from different distributions *J*_2_, *J*_3_, …). The shedding begins *k* ∈ *K* time prior to the end of the interval. There is a delay *d* ∈ *D* from the screening event prior to isolating an infectious individual, reflecting the turnaround time of the test.

There is a variable probability of transmission (relative infectivity) during the asymptomatic/presymptomatic shedding period. At its most general, this can be expressed as a normalized force of infection *g*(*t*, *τ*), satisfying

g(t,τ)=0,t<0 or t>τ

and

$$\int_{0}^{\infty }\int_{0}^{\tau }g(t,\tau )f_{M}(\tau )\;dt\;d\tau =1.$$

Here, *f*_*M*_ is the probability density function for the shedding duration *M*, and *t* and *τ* parameterize the position within the shedding period and the total duration of shedding, respectively. With this definition, in the absence of screening the total normalized infectivity is 1. The total normalized infectivity with screening and isolation is therefore expressed as a proportion. This proportion can be used as a coefficient to determine the effect of screening on the reproduction number *R*. In this paradigm, relative infectivity depends upon both position in the shedding period and total duration of shedding: groups with different shedding durations can have different total infectivities. *g* can be interpreted as a composite reflecting the average shedding profile of an individual. The most obvious way in which *g* can be seen to be a composite is that if the same shedding period can represent both asymptomatic and presymptomatic shedding, then *g* represents a composite of the two normalized by their respective probabilities.

Next, we reflect that screening does not pick up all infectious individuals by introducing a probability of detection *p*_*d*_(*t*, *τ*) ∈ [0, 1]. This probability is allowed to depend on the total duration of shedding (i.e. the total duration of shedding *τ* is used to parameterize how easy it is to detect viral shedding at a given time *t*). It represents a composite of the probability that an individual presents for screening, the sensitivity of the screening test, and the probability that the individual isolates following a positive test. For nucleic acid amplification testing for SARS-CoV-2, both *g*(*t*, *τ*) and *p*_*d*_(*t*, *τ*) are related to viral shedding, and hence they are related to each other. However, shedding of non-viable RNA later in the course of infection means that *p*_*d*_(*t*, *τ*) tapers more slowly than *g*(*t*, *τ*) over time.

We now proceed to derive a generalized expression for the relative infectivity in the presence of a given screening regimen. First, we derive an expression for *f*_*K*_(*x*), the density function for the amount of time before the first screen that shedding starts. We assume a constant hazard for the commencement of shedding over time. This assumption is valid as long as screening maintains an equilibrium of infections (there is not a large growth or contraction in infection prevalence between successive screens), and as long as the time of onset of infectiousness can be assumed independent between individuals (true in all but very small populations). Note, however, that the screening interval length *j* can vary. This means that the time before the first screen that shedding starts, *K*, is not simply distributed uniformly across the sampled screening interval *j*, but needs to be conditioned upon the probability that shedding begins in a particular interval. For *J*^′^ the distribution of the screening interval length in which the shedding begins, we have

$$f_{J^{\prime }}(x)\propto xf_{J}(x),$$

and by integrating both sides with respect to *x* we can derive the constant of proportionality and conclude that

$$f_{J^{\prime }}(x)=\frac{xf_{J}(x)}{E(J)}.$$

It then follows that

$$\begin{array}{rl} \;f_{K}(x) & =\int_{0}^{\infty }\frac{\;f_{J^{\prime }}(y)}{y}I(x\in (0,y))\;dy\\ & =\int_{x}^{\infty }\frac{\;f_{J}(y)}{E(J)}\;dy\\ & =\frac{1-F_{J}(x)}{E(J)}. \end{array}$$

Now that we have a density function for the time from the start of shedding to the next screen, we can write down a density function for the time from the start of shedding to the time an individual with a positive screen result can be isolated. This is simply the convolution

$$\begin{array}{rl} \;f_{K+D}(x) & =\int_{0}^{\infty }f_{K}(y)f_{D}(x-y)\;dy\\ & =\int_{0}^{\infty }\frac{1-F_{J}(y)}{E(J)}f_{D}(x-y)\;dy. \end{array}$$

This convolution is independent of the total shedding duration. However, its sampled value can exceed the total shedding duration, that is, shedding can have ended before it is possible to act on a screening result.

We can now write down our first, simplified expression for averted infectivity. In this simplified case, a screen always identifies an infectious individual, and that individual then perfectly isolates. Conditioned upon a total shedding time *τ*, the normalized infectivity averted is

$$\int_{0}^{\infty }f_{K+D}(t)\int_{\min(t,\tau )}^{\tau }g(x,\tau )\;dx\;dt,$$

where the min reflects the possibility of shedding having ended prior to the screen.

Using *g*(*x*, *τ*) = 0 for *x* > *τ* we can expand the components of the integral to give a normalized infectivity averted for shedding time *τ* of

$$A_{1}(\tau )=\int_{0}^{\tau }\int_{0}^{\infty }\int_{t}^{\tau }\frac{1-F_{J}(y)}{E(J)}f_{D}(t-y)g(x,\tau )\;dx\;dy\;dt.$$

The total normalized infectivity averted from one infectivity-abolishing screen is therefore

$$\begin{array}{rl} A_{1} & =\int_{0}^{\infty }A_{1}(\tau )f_{M}(\tau )\;d\tau \\ & =\int_{0}^{\infty }\int_{0}^{\tau }\int_{0}^{t}\int_{t}^{\tau }f_{M}(\tau )\left(\frac{1-F_{J}(y)}{E(J)}\right)f_{D}(t-y)g(x,\tau )\;dx\;dy\;dt\;d\tau . \end{array}$$

Next, we consider the situation where the probability that a screen abolishes infectivity need not be 1. In this case, conditioned upon an overall probability *p*_∞_ of *ever* detecting infection in an individual given arbitrarily many tests over arbitrarily short testing intervals, a successful screen at time *y* occurs with probability *p*(*y*, *τ*) and unsuccessful screens happen at time *a*_(1,1)_ = *a*_1_, *a*_(1,2)_ = *a*_1_ + *a*_2_, *a*_(1,3)_ = *a*_1_ + *a*_2_ + *a*_3_, … < *y* with probabilities 1 − *p*(*a*_(1,1)_, *τ*), 1 − *p*(*a*_(1,2)_, *τ*), 1 − *p*(*a*_(1,3)_, *τ*), …. (In our earlier notation, *p*_*d*_(*t*, *τ*) = *p*_∞_*p*(*t*, *τ*).) Note that the length of shedding in the interval before the first screen is still described by the random variable *K*, but in subsequent intervals shedding occurs across the entire interval unless it finishes altogether, so is described by *J*_*i*_, *i* ≥ 2. Electronic supplementary material, figure S3, gives a schematic of this set-up to illustrate the notation used. Testing turnaround time is only applicable after the last, successful, screen. (If turnaround time is highly variable, it is in principle possible to have two positive screens with the result of the second available before the first. In practice, most laboratory testing is first in, first out, so we neglect this possibility.)

For any given number of screens prior to successful detection, we can express the infectivity averted by taking a convolution of the distributions of testing times as an extension to the expression for *f*_*K*+*D*_(*x*). This yields a general expression for the distribution of the time from beginning of shedding (with total shedding length *τ*) to isolation of an individual with a positive test result:

$$\begin{array}{rl} \;f_{T}(t|\tau ) & =p_{\infty }\int_{0}^{t}p(y,\tau )f_{K}(y)f_{D}(t-y)\;dy\\ & \;+p_{\infty }\int_{0}^{t}\int_{0}^{y}p(y,\tau )(1-p(a_{1},\tau ))f_{K}(a_{1})f_{J_{2}}(y-a_{1})f_{D}(t-y)\;da_{1}\;dy\\ & \;+p_{\infty }\int_{0}^{t}\int_{0}^{y}\int_{0}^{y-a_{1}}p(y,\tau )(1-p(a_{1},\tau ))(1-p(a_{\left(1,2\right)},\tau ))\\ & \;\;\times f_{K}(a_{1})f_{J_{2}}(a_{2})f_{J_{3}}(y-a_{\left(1,2\right)})f_{D}(t-y)\;da_{2}\;da_{1}\;dy+\cdots \\ & \;+p_{\infty }\int_{0}^{t}\int_{0}^{y}\int_{0}^{y-a_{1}}\int_{0}^{y-a_{\left(1,2\right)}}\ldots \int_{0}^{y-a_{\left(1,n-2\right)}}p(y,\tau )\left(\prod_{i=1}^{n-1}(1-p(a_{\left(1,i\right)},\tau ))\right)\\ & \times f_{K}(a_{1})\left(\prod_{i=2}^{n-1}f_{J_{i}}(a_{i})\right)f_{J_{n}}(y-a_{\left(1,n-1\right)})f_{D}(t-y)\;da_{n-1}\;da_{n-2}\ldots \;da_{1}\;dy\\ & \;+\cdots . \end{array}$$

The total infectivity averted from screening is therefore

$$\begin{array}{rl} A & =\int_{0}^{\infty }\int_{0}^{\infty }\int_{\min(t,\tau )}^{\tau }f_{M}(\tau )f_{T}(t|\tau )g(x,\tau )\;dx\;dt\;d\tau \\ & =p_{\infty }\sum_{n=1}^{\infty }A_{n}, \end{array}$$

where

$$\begin{array}{rl} & A_{1}=\int_{0}^{\infty }\int_{0}^{\tau }\int_{0}^{t}\int_{t}^{\tau }f_{M}(\tau )p(y,\tau )\left(\frac{1-F_{J}(y)}{E(J)}\right)f_{D}(t-y)g(x,\tau )\;\;dx\;dy\;dt\;d\tau ,\\ & A_{2}=\int_{0}^{\infty }\int_{0}^{\tau }\int_{0}^{t}\int_{0}^{y}\int_{t}^{\tau }f_{M}(\tau )p(y,\tau )(1-p(a_{1},\tau ))\left(\frac{1-F_{J}(a_{1})}{E(J)}\right)\\ & \;\;\times f_{J_{2}}(y-a_{1})f_{D}(t-y)g(x,\tau )\;dx\;da_{1}\;dy\;dt\;d\tau ,\\ & A_{n}=\int_{0}^{\infty }\int_{0}^{\tau }\int_{0}^{t}\int_{0}^{y}\int_{0}^{y-a_{1}}\int_{0}^{y-a_{\left(1,2\right)}}\ldots \int_{0}^{y-a_{\left(1,n-2\right)}}\int_{t}^{\tau }f_{M}(\tau )p(y,\tau )\\ & \;\;\times \left(\prod_{i=1}^{n-1}(1-p(a_{\left(1,i\right)},\tau ))\right)\left(\frac{1-F_{J}(a_{1})}{E(J)}\right)\left(\prod_{i=2}^{n-1}f_{J_{i}}(a_{i})\right)\\ & \;\;\times f_{J_{n}}(y-a_{\left(1,n-1\right)})f_{D}(t-y)g(x,\tau )\;dx\;da_{n-1}\;da_{n-2}\ldots \;da_{1}\;dy\;dt\;d\tau . \end{array}$$

These integrals are high-dimensional, but we have successfully implemented code to calculate them in Mathematica [13], using its numerical integration algorithms. (We use the global adaptive numerical integration method unless otherwise stated, but have implemented other methods in the code for cross-checking.) The finite limits on many of the integrals, plus the ability to truncate the infinite integral when the probability of shedding is low, mean that the first few terms in the sum are computationally tractable in full.

When successive screens are close together, so that multiple terms in the sum become relevant, performing the full multidimensional integrals becomes computationally intractable. However, when the time between one screening opportunity and the following opportunity is very similar for all individuals eligible for screening, the above convolutions can be markedly simplified. The appropriate simplification is the approximation $f_{J_{i}}(t)=\delta (t-t_{i})$ for *i* ≥ 2, where *δ* is the Dirac delta and *t*_*i*_ are fixed intervals between successive screens (which may differ from each other). This allows reduction of all the *A*_*i*_ to four-dimensional integrals: *A*_1_ is unchanged, and we have

$$\begin{array}{rl} A_{2} & =\int_{t_{2}}^{\infty }\int_{t_{2}}^{\tau }\int_{t_{2}}^{t}\int_{t}^{\tau }f_{M}(\tau )p(y,\tau )(1-p(y-t_{2},\tau ))\left(\frac{1-F_{J}(y-t_{2})}{E(J)}\right)\\ & \;\times f_{D}(t-y)g(x,\tau )\;dx\;dy\;dt\;d\tau \end{array}$$

and

$$\begin{array}{rl} & A_{n}=\int_{t_{\left(2,n\right)}}^{\infty }\int_{t_{\left(2,n\right)}}^{\tau }\int_{t_{\left(2,n\right)}}^{t}\int_{t}^{\tau }f_{M}(\tau )p(y,\tau )\\ & \times \left(\prod_{i=2}^{n}(1-p(y-t_{\left(i,n\right)},\tau ))\right)\left(\frac{1-F_{J}(y-t_{\left(2,n\right)})}{E(J)}\right)f_{D}(t-y)g(x,\tau )\;dx\;dy\;dt\;d\tau , \end{array}$$

where *t*_(*i*,*j*)_ is defined analagously to *a*_(*i*,*j*)_. In a programme undertaking many screens at short intervals, this approximation is likely to prove more accurate than early truncation of a series containing the full, multiple-integral, terms. We note that the approximation is better as *p*(*t*, *τ*) is more smooth, although the variability in the time to initial sampling means that even when *p*(*t*, *τ*) is rapidly varying, the approximation is still good as long as the intervals between successive screens are similar.

### 4.1. Model parameters

To evaluate the effects of different screening scenarios, we set parameters of the model according to estimates for SARS-CoV-2 or the parameters we wished to evaluate. These are described in detail in the electronic supplementary material.

## Data accessibility

The datasets supporting this article have been uploaded as part of the supplementary material, and include machine-readable versions of data from Zhao *et al.* [14]; Xia *et al.* [15]; Lauer *et al.* [16]; Hu *et al.* [17]; Arons *et al.* [18]; Rivett *et al.* [1]; Ferretti *et al.* [19]; Zhao *et al.* [8]; PHE Porton Down & University of Oxford SARS-CoV-2 LFD test development and validation cell [9].
